# Mapping the climatic suitable habitat of oriental arborvitae (*Platycladus orientalis*) for introduction and cultivation at a global scale

**DOI:** 10.1038/srep30009

**Published:** 2016-07-21

**Authors:** Guoqing Li, Sheng Du, Zhongming Wen

**Affiliations:** 1 State Key Laboratory of Soil Erosion and Dryland Farming on Loess Plateau, Northwest A&F University, Yangling 712100, China; 2 Institute of Soil and Water Conservation, Chinese Academy of Sciences and Ministry of Water Resources, Yangling 712100, China

## Abstract

Oriental arborvitae (*Platycladus orientalis*) is an important afforestation and ornamental tree species, which is native in eastern Asian. Therefore, a global suitable habitat map for oriental arborvitae is urgently needed for global promotion and cultivation. Here, the potential habitat and climatic requirements of oriental arborvitae at global scale were simulated using herbariums data and 13 thermal-moisture variables as input data for maximum entropy model (MaxEnt). The simulation performance of MaxEnt is evaluated by ten-fold cross-validation and a jackknife procedure. Results show that the potential habitat and climate envelop of oriental arborvitae can be successfully simulated by MaxEnt at global scale, with a mean test AUC value of 0.93 and mean training AUC value of 0.95. Thermal factors play more important roles than moisture factors in controlling the distribution boundary of oriental arborvitae’s potential ranges. There are about 50 countries suitable for introduction and cultivation of oriental arborvitae with an area of 2.0 × 10^7^ km^2^, which occupied 13.8% of land area on the earth. This unique study will provide valuable information and insights needed to identify new regions with climatically suitable habitats for cultivation and introduction of oriental arborvitae around the world.

*Platycladus orientalis*, commonly known as oriental arborvitae, Chinese arborvitae, and oriental cedar, is the only species in its genus within the family of Cupressaceae^1^. It is naturally widespread across northwestern China, Korea, and Far East Regions of Russian. It is also a naturalized species in Europe, north American, eastern Africa and some Asian countries (Japan, India and Iran) where it has been introduced historically, most likely by settlers as a afforestation and greening species. There are some relic populations of this species existing in Central Asia, which are often supposed to be along the Silk Road and are not natural populations^2^. Usually, oriental arborvitae is used as a reforestation species in vulnerable areas due to its resistance to cold, dry, and salt environment^3^. Oriental arborvitae is also used as an ornamental tree species, especially around Buddhist temples in China, which is mainly associated with long life, unchanging evergreen leaves and vitality in Buddhist thought in China^4^. There are many benefits of oriental arborvitae to human, for example, its wood can be used for architecture, furniture, farm implements and its leaves, barks and seeds can be used as a Chinese herbal medicine for treatments of insomnia, hemostasis, blood stasis, and uneasiness of mind and body^1^.

As a multi-purpose species, especially a reforestation species, oriental arborvitae has been investigated mainly in the technical aspect of plantation development, including breeding, management, and plantation, etc. For example, Chen *et al.*^5^ have studied the geographical variation of seedling traits across China and suggested that the quality of seedlings from southeast of China is better than that from northwest of China. Xu and Han^6^,^7^ have suggested the main pest control measures for oriental arborvitae and Du *et al.*^8^ reviewed the advances in plantation silviculture for oriental arborvitae. Hu *et al.*^9^ have evaluated the impact of future climate change on distribution of oriental arborvitae in China. These studies have provided the important theoretical basis for the cultivation of oriental arborvitae in China. However, the climatically suitable habitat and climatic response characteristics of oriental is poor understood from a global perspective, which limits the introduction and cultivation of this species around the world and maximizing its ecological and cultural value. Therefore, a global suitable habitat map for oriental arborvitae is urgently needed.

In recent years, species distribution modeling (SDM) has been widely used to estimate ecological requirement of particular species and to characterize and map the spatial distribution of habitat occupied by species at a landscape scale^10^,^11^,^12^,^13^. The principle of SDM is related to Hutchinson super-volume theory, and emphasizes the ecological requirement of species, especially the abiotic factors controlling species distribution^14^,^15^. In general, many climatic factors were used as predicted variables when simulating species potential distribution at large scale with coarse resolution. According to the predicted map, we can depict the climatic niche and response curves of species. Booth^16^,^17^ suggested that the obtained information is very useful for the cultivation and reintroduction of plant species to a new region. Currently, there are many algorithms in SDM technique, but Elith *et al.*^18^ have shown that maximum entropy model (MaxEnt) is one of the best method among dozens of algorithms (including climatic envelop method, distance based method, genetic method, MaxEnt, etc.). Philiple *et al.*^19^,^20^ suggested some merits of MaxEnt, like it only need presence data, and show good performance with much fewer number of presence data compared to presence/absence data driven models. It could also treat continuous variables as well as categorical variables. What’s more, Elith *et al.*^21^ have expanded the ability of the MaxEnt model by increasing the limiting factor mapping and similar surface mapping for range-shifting species, which could tell us how the credibility and reliability of climatic habitat suitability and why species could (not) distribute there.

The aim of this work was to simulate the potential distribution area of oriental arborvitae and figure out the significant climatic factors of the species at a global scale. Firstly, we collected the occurrence records from many herbarium database and publication resources; Secondly, we also collected a set of climatic variables including 13 factors come from Bioclim system^22^, Kira system^23^, and Holdridge system^24^. Finally, we used the occurrence records and climatic factors as input data of MaxEnt model to simulate the potential distribution area of oriental arborvitae according to workflow that we have designed in advance^25^,^26^. This study is mainly concerned with the following objects: (1) identifying climatically suitable habitats for oriental arborvitae at a global scale; (2) mapping limiting climatic factors and estimating climatic thresholds (niche) of oriental arborvitae; (3) determining suitable areas for the introduction and cultivation of oriental arborvitae around the world. The answers to the three questions could not only enhance our understanding of the causes of its distribution range, but also provide references for the promotion of oriental arborvitae around the world.

## Results

### The current and potential distribution of oriental arborvitae

**Figure 1:**
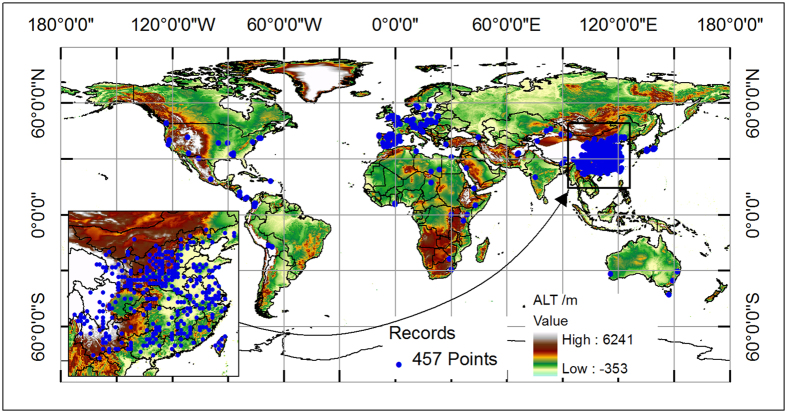
Global spatial distribution of occurrence records (457 points) of oriental arborvitae with a grid cell of 0.5° × 0.5° resolution and spatial distribution of altitude around the world (altitude layer come from website: http://www.worldclim.org/). It shows that the altitude distribution of oriental arborvitae from 0 to 4526 m (1st. Qu. 128 m, Median 492 m, Mean 795 m, and 3rd Qu. 1132 m). The whole map is generated by using the tool of ArcGIS 9.3 (ESRI, Redlands, CA, USA, http://www.esri.com/).

Based on the occurrence records of oriental arborvitae in the GBIF and CVH databases, the map of the current distribution was shown in Fig. 1. Oriental arborvitae occurs mainly in 23 countries: Asia (China, Japan, India, Afghanistan), Europe (United Kingdom, France, Spain, Germany, Poland, Austria), North and Central America (United States, Mexico, Panama, Nicaragua, Costa Rica), South America (Colombia, Bolivia), Africa (Libya, Ghana, Kenya, Ethiopia, South Africa), and other regions (Australia). Most of records were collected in Europe and Asia countries.

**Figure 2:**
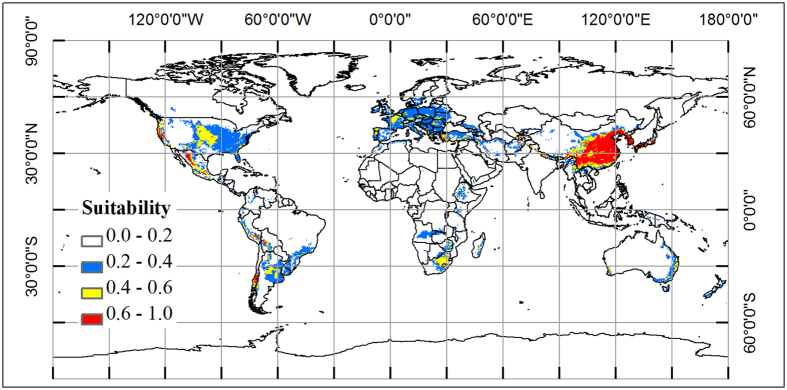
Spatial distribution map of potential area for oriental arborvitae around the world, which is produced by MaxEnt v3.3 (http://www.cs.princeton.edu/~schapire/maxent/), with a variation of each cell 0–0.11. Blue color represents marginal habitat, orange color represents median habitat, and red color represents core habitat. The whole map is generated by using the tool of ArcGIS 9.3 (ESRI, Redlands, CA, USA, http://www.esri.com/).

An average probability of climatic suitable habitat map was produced from 10-fold cross validation by MaxEnt model with 13 climatic variables (Fig. 2). According to the map, the potential distribution area of oriental arborvitae (probability threshold >0.2) mainly locate in the following 50 countries (Table 1), which belong to Asia (11), Europe (22), North and Central America (2), South America (7), Africa (6), and Oceania (2) regions. We found that the climatic suitable area (probability threshold >0.2) was about 2.0 × 10^7^ km^2^, which accounted for 13.8% of land area around the world.

Based on the probability value, we divided the habitat into three classes: marginal area (0.2–0.4), median area (0.4–0.6), and core area (0.6–1.0). The structure of different level habitat was shown in Fig. 2. The core area is about 3.4 × 10^6^ km^2^, which is mainly located in China, Korea, Japan, Turkey, United States, Mexico, Peru, Bolivia, and Chile. The climate condition of oriental arborvitae around the world and each region could be seen in Supplementary Material Table S1–7. We found that the most suitable climate condition was −41.7–0 °C of CI, 54–235.8 °C of WI, 62–736 mm of PWM, 3.2–20.1 °C of AMT, 8–143% of PSD, and 411–3272 mm of AP.

### Model performance and importance of climatic factors

The goodness-of-fit test of MaxEnt was evaluated by ten-fold cross validation method and the relative contribution of each climatic factor was evaluated by a jacknife test. The accuracy of the simulation results were shown in Supplementary Material Figure S1. It showed that MaxEnt model predictions were highly accurate with a mean training AUC of 0.935 (ranging from 0.932 to 0.936) and test AUC of 0.918 (ranging from 0.903–0.934). The test AUC was close to 1, which indicated that the model performed better than random and therefore showed the high accuracy of the model. The coefficient of variation was only 1.12% among ten predictions, indicating that the 10-fold cross-validation method did not affect the accuracy of MaxEnt model simulation.

**Figure 3:**
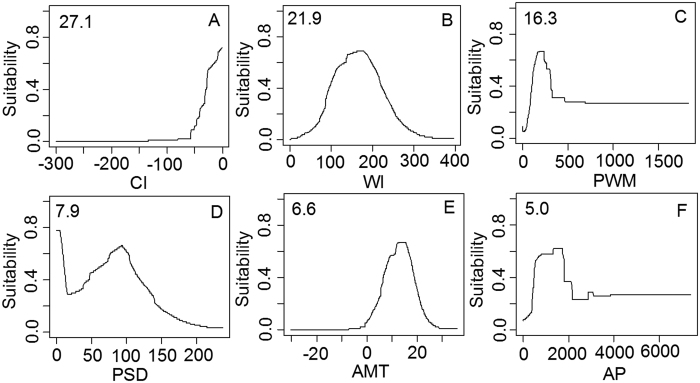
The response curves of climatic suitability for six dominant climatic factors based on MaxEnt model and their relative importance shown in the upper-left corner of each subplot. (**A**) coldness index (CI, °C); (**B**) warmth index (WI, °C); (**C**) precipitation of wettest month (PWM, mm); (**D**) precipitation of seasonality (PSD, %); (**E**) annual mean temperature (AMT, °C); (**F**) annual precipitation (AP, mm).

The relative contribution of each climatic factor was shown in Supplementary Material Figure S2. We found that CI, WI, PWM, PSD, AMT, and AP were the most important climatic factors determining the distribution of oriental arborvitae. These six factors could explain 84.8% of the variance, and could be divided into thermal group (CI, WI, AMT, 55.6%) and moisture group (PWM, PSD, AP, 29.2%). It appeared that thermal condition was more important than humidity condition in controlling the geographical ranges of plant species. The response curves of oriental arborvitae under the first six most important climatic factors were shown in Fig. 3. It was clear that unimodal relationships existed between the habitat suitability value and AMT, WI, PWM, PSD, while the CI showed an exponential relationship with the habitat suitability value. The response peak in oriental arborvitae habitat suitability for the CI was at 0 °C, for the WI, it was at 170 °C, for the AMT, it was at 14 °C, for the PWM, it was at 210 mm, for the PSD, it was at 92%, and for the AP, it was at 1500 mm.

## Discussion

The species distribution modeling is a very useful tool to predict the potential distribution of species^14^,^10^. Currently, many groups of species have been simulated by SDM for the purposes of protection, cultivation, and disease prevention. Such as endanger species, crops, pathogenic bacteria, etc. Here, we used oriental arborvitae, a multi-purpose species, especially an afforestation species, as a target species, to simulate its potential distribution based on herbarium data and climate data by MaxEnt and GIS tools. Our results show that the climatically suitable habitat of oriental arborvitae can be accurately predicted at global scale. The average AUC value of 10 cross-validation has reached the very good level (greater than 0.9). As far as we known, few study has been focused on simulation of the climatic suitable habitat of oriental arborvitae around the world. This work benefits from the development of network techniques and database construction. Global Biodiversity Information Facility^27^, Atlas of Living Australia^28^ and Chinese Virtual Herbarium^29^ represents major advance in global biodiversity databases and ecological modeling, which have been applied in decision making for tree introduction and cultivation around the world for several years^17^.

**Figure 4:**
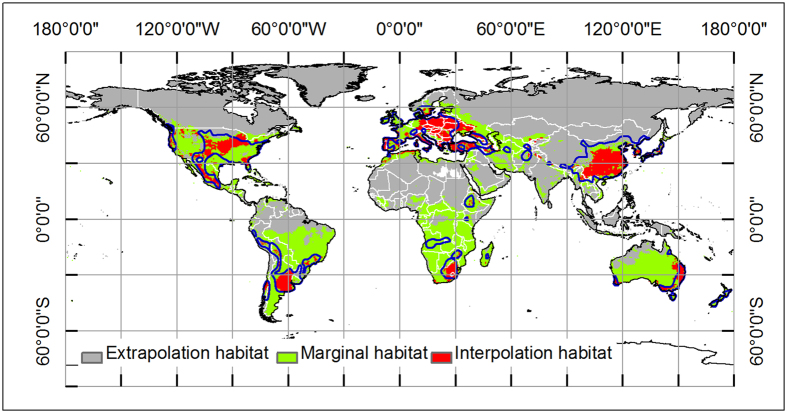
Multivariate environmental similarity surface (MESS) map of novel habitat, which is produced by MaxEnt v3.3 (http://www.cs.princeton.edu/~schapire/maxent/). Coarse blue polygon represents potential distribution range of oriental arborvitae using threshold of 0.2. Red color represents interpolation habitat (positive value), green color represents marginal habitat (near zero), and grey color represents extrapolation habitat (negative value). The whole map is generated by using the tool of ArcGIS 9.3 (ESRI, Redlands, CA, USA, http://www.esri.com/).

The herbarium data used in simulating process was not only from native habitat ranges, but also from cultivation regions. Therefore, we infer that the climatic condition and species response curves we simulated consist of the fundamental niche of oriental arborvitae as Booth *et al.*^30^,^31^ have demonstrated that information from outside of the native range could imply fundamental niche of plant species. The simulation of climatic suitable area is equivalent to the potentially occupied area, which was also referred to as the inviable distribution area by Peterson *et al.*^12^ according to the conceptual model for explaining the relationship between species distribution model and species distribution area. This means species dispersal ability are unlimited and species interaction are absent. Besides, Elith *et al.*^21^ have enhanced the infer ability of MaxEnt model about where is the novel habitat for a particular species. Our simulating results suggest that most of the suitable climatic habitats belong to interpolation habitat for oriental arborvitae at global scale (Fig. 4), which means that the credibility and reliability of the map are high.

**Figure 5:**
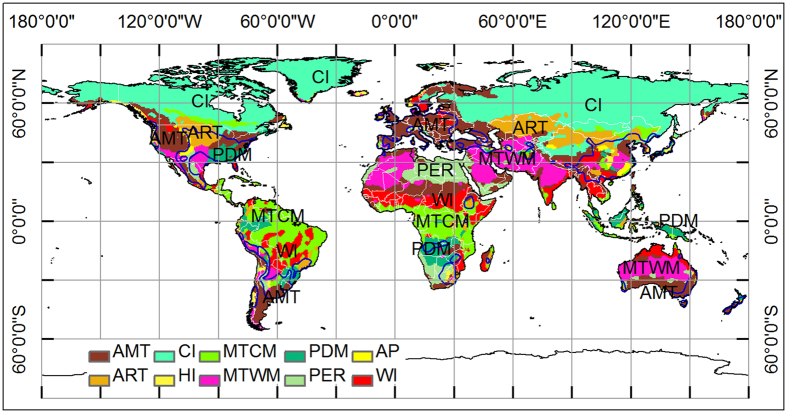
Spatial distribution of limiting factors for oriental arborvitae around the world, which is produced by MaxEnt v3.3 (http://www.cs.princeton.edu/~schapire/maxent/). The limiting factors at any grid cell is generated from MESS analysis by finding the variable with the smallest MESS value. LFM analysis brings insight into which climatic factors mostly limit physiological and ecological processes in each grid cell in target areas. Coarse blue polygon represents potential distribution range of oriental arborvitae using threshold of 0.2. coldness index (CI), warmth index (WI), annual mean temperature (AMT), annual precipitation (AP), annual range of temperature (ART), precipitation of driest month (PDM), mean temperature of the warmest month (MTWM), mean temperature of the coldest month (MTCM), humidity index (HI), and potential evapotranspiration rate (PER). The whole map is generated by using the tool of ArcGIS 9.3 (ESRI, Redlands, CA, USA, http://www.esri.com/).

Our simulating results have shown that thermal factors (CI, WI, AMT) play more important role than moisture factors (PWM, PSD, AP) in controlling the potential distribution of oriental arborvitae at global scale. There exist a complex relationship between suitability of oriental arborvitae and climatic factors, including exponential function (e.g. CI) and Gaussian function (e.g. WI). Limiting factors mapping analysis brings insight into which climatic factors mostly limit physiological and ecological processes in each grid cell in target areas (Fig. 5). If there are several unfavorable factors in a region, the factor with the smallest multivariate environmental similarity surface (MESS) value will be selected as candidate factor for limiting factors mapping. For example, MTWM in the core area of central China means the same thing as that in Middle East and North Africa in the mathematical perspective, which means MTWM is the most climatic limiting factors in all 13 climatic factors. But, MTWM in the core area of central China is very different from that in Middle East and North Africa from the biological perspective, which means MTWM affects the growth and development of oriental arborvitae in central China, while MTWM affects the survival and occurrence of oriental arborvitae in Middle East and North Africa.

Chuine^32^ is convinced that phenology determines the boundary of species distribution range from the perspective of mechanism. Therefore, we infer that northern boundary of oriental arborvitae’s ranges appears to be formed mainly due to the inability of this species to undergo full fruit maturation as insufficient heat accumulation in a cold-climate region, while the southern limit appears to be formed due to the inability of this plant species to flower or unfold leaves as excess heat accumulation in a hot-climate region. The limiting factor map (Fig. 5) also shows that moisture factors (e.g. AP, PWM) are less important than thermal factors from the perspective of controlling distribution areas. The key climatic factors affecting the distribution of oriental arborvitae are similar with those of *Fagus* spp^33^,^34^ and these species are both primarily affected by thermal-related factors (CI, WI, and AMT for oriental arborvitae; growing season warmth, CI and MTCM for genus species of *Fagus*). However, the distribution of oriental arborvitae is different from that of *Pinus tabulaeformis*^35^ and *Quercus wutaishanica* 36. The latter two species are dominant tree species of forest in Northern China and they are mainly controlled by hydrological-thermal-related variables (PWM, MTCM and ABT for *P. tabulaeformis*, precipitation of coldest quarter and MTWM for *Q. wutaishanica*). Such a combination of water-heat condition is dominated by the unique East Asian monsoon climate system^35^,^36^, which makes *P. tabulaeformis* and *Q. wutaishanica* endemic species in China. The southern distribution boundaries of *P. tabulaeformis* and *Q. wutaishanica* cannot beyond the dividing line between subtropical zone and warm temperate zone in China (Qinling Mountain-Huaihe River line). In addition, the climatic envelope of *P. tabulaeformis* has been projected to the world map (provided in Supplementary Material in Li *et al.*^35^), and the results demonstrate that only a small area of climatic suitable habitat for *P. tabulaeformis* exists outside of China (Midwestern USA, central Asia, and Korean peninsula). This means that *P. tabulaeformis* can only be widely cultivated and planted in China due to climatic restrictions.

As a valuable afforestation species, the ecological traits of oriental arborvitae (native in East Asia) are very similar to those of black locust (*Robinia pseudoacacia*, native in North America), which is also a globally cultivated and introduced species^25^. For example, they share the similar spatial distribution of climatic suitable habitat and their climatic fitness show similar response curves to climatic factors. Thermal condition is more important than humidity condition in controlling both species’ geographical ranges. However, the biological traits of oriental arborvitae are different from those of black locust. After 300 years of introduction and cultivation, black locust has shown the characteristics of invasive ability^37^. Currently, no technique is available to provide effective control of black locust invasions^38^. Although oriental arborvitae has been cultivated and introduced worldwide for several hundred years, there is no report on its invasion to native forests or other vegetation. This can be explained from the other side: oriental arborvitae is a kind of species with ecological security to environment and is also a species with economically valuable to society, which will make it a great potential application value in the future.

Our simulated climatic suitable map of oriental arborvitae has many uses. For example, we can divide the climatic habitats into three classes: core area, medial area, and marginal area. Then, we can easily conclude that the most suitable climate condition of oriental arborvitae is −41.7–0 °C for CI, 54–235.8 °C for WI, 62–736 mm for PWM, 3.2–20.1 °C for AMT, 8–143% for PSD, and 411–3272 mm for AP. By comparing the distribution of herbarium data and the potential area (Figs 1 and 2), we can find countries with suitable habitats for the introduction of oriental arborvitae for ornamental and afforestation purposes. These countries include China, Korea, Japan, Turkey, United States, Mexico, Peru, Bolivia, Chile, United Kingdom, France, Germany, Spain, Portugal, Italy, Australia, New Zealand, Morocco, Ethiopia, Kenya, South Africa, etc. (detailed information could be seen in Table 1). These regions mainly located at temperate zone without dry season (Cf, 32.3%), temperature zone with dry winter (Cw, 14.8%), cold zone without dry season (Df, 20%), arid steppe zone (Bs, 12.6%) according to the Koppen-Geiger climate classification of the world^39^. Based on the climatic suitable map of oriental arborvitae, we can plan the plantation of oriental arborvitae combined with recently remote sensing data at global scale and national scale. We can also use the climatic response curves to calculate the climatic fitness of oriental arborvitae at any location with local climate station data. Then, management strategies of oriental arborvitae forest could be constructed appropriately.

According to the Global Forest Resources Assessment 2015^40^, net loss of forest area between 1990 and 2015 occurred in tropical countries (e.g. Brazil, Indonesia, Zaire), while net gain in forest area happened in temperate countries (e.g. China, United States). There has been relatively little change in forest area of the boreal and subtropical countries (e.g. Canada, Mongolia, Saudi Arabia). Generally, the driving forces of world’s forest area change mainly came from human activate, such as deforestation and reforestation, which were usually manoeuvred by local governments. As an excellent afforestation tree species, oriental arborvitae can grow widely around the world, and the climatically suitable habitat of oriental arborvitae occupied 13.8% of land area on the earth, which is mainly located at temperate regions. We recommended that oriental arborvitae should be considered as a candidate tree for countries carrying out afforestation program, such as China, France, United States, Argentina, South Africa. Currently, Chen *et al.*^5^ have shown that the quality of seedlings from southeast of China are better than those from northwest of China. Therefore, seedlings from southeast of China are very suitable for the promotion around the world. We believe that oriental arborvitae, as a very promising tree species, has great potential in plantation around the world in an era of human-induced forest degradation and global climate change.

## Materials and Methods

### Target species and occurrence data

Oriental arborvitae is an evergreen and slow-growing tree of about 15–20 m tall. It is suited to the dry-cold and wet-warm climate condition. In the current research, three resources were used to search for the present sites of oriental arborvitae in the world: (1) Global Biodiversity Information Facility (GBIF)^27^ (a free and open access biodiversity database that integrates existing worldwide biodiversity data to form a user-oriented global biodiversity service network); (2) Chinese Virtual Herbarium (CVH)^29^ (a free and open access database that integrates the herbarium data of national natural museums from 14 institutes of China); (3) published literature^5^,^41^,^42^. Finally, we got 945 occurrence points (632 from GBIF, 235 from CVH, and 78 from published literatures). Previous reports have said that there may have been a sampling bias or error at a fine resolution in the GBIF and CVH occurrence records, which would produce models of lower quality rather than that of higher quality^43^,^44^. So we assigned the occurrence point to a coarse resolution (0.5° × 0.5°) according to previous studies with a spatial resolution between 50 km × 50 km and 200 km × 200 km. Detailed information of the workflow could be seen in Li *et al.*^25^,^26^. Finally, we converted the collected 945 points at fine resolution to 457 points at 0.5° × 0.5° resolution in a world map (Fig. 1), which shows the altitude distribution of this species from 0 to 4526 m (1st. Qu. 128 m, Median 492 m, Mean 795 m, and 3rd Qu. 1132 m).

### Climatic variables

Climatic factors play much more important role in determining the potential distribution of species than soil factors and topography factors at large scale^45^. So climatic factors are widely used as predicting variables to simulate potential distribution ranges of species at global scale with coarse resolution. Currently, there are many climatic systems to characterize global climate niche, such as Holdridge system^24^ (including annual precipitation, potential evapotranspiration rate, annual biotemperature), Kria system^23^ (including warmth index, coldness index, humidity index), and Bioclim system^22^ (including 19 BIOCLIM variables). Integrating these 23 climatic factors (instead of 24 climatic factors because of annual precipitation occurring simultaneously in Holdridge system and BIOCLIM system) as a new set of predicting variables has been demonstrated a good choice for simulating species potential distribution area.

An excess of climatic variables can cause overfitting, so we selected 13 of the 23 climatic variables related to temperature, precipitation, growing degree days, thermal and moisture factors (6 Holdridge-Kira variables and 8 BIOCLIM variables, annual precipitation occurring simultaneously in Holdridge system and BIOCLIM system), because they were usually more important for limiting species distribution, especially climate extremes and seasonality variables. Temperature variables include annual mean temperature (AMT), max temperature of the warmest month (MTWM), min temperature of the coldest month (MTCM), and annual range of temperature (ART = MTWM-MTCM). Precipitation variables include annual precipitation (AP), precipitation of wettest month (PWM), precipitation of driest month (PDM), and precipitation of seasonality (PSD = Monthly coefficient of variation of precipitation). Growing degree days is represented by annual biotemperature [ABT = ∑*T*/12, *T* is mean monthly temperature]. Thermal variables including warmth index [WI = ∑(*T* − 5), *T* > 5 °C, *T* is mean monthly temperature], coldness index [CI = ∑(*T* − 5), *T* < 5 °C, *T* is mean monthly temperature). Moisture variables include potential evapotranspiration rate (PER = 58.93 × ABT/AP, ABT is annual biotemperature, AP is annual precipitation), humidity index (HI = AP/WI, AP is annual precipitation, WI is warmth index). The climate layers were generated based on thin-plate smoothing splines with latitude, longitude, altitude, and monthly temperature and precipitation records from averages of 50-year climate station records (1950–2000) around the world^22^,^46^.

### Simulation process and evaluation procedure

We used a famous modeling method called maximum entropy algorithm or MaxEnt which has been found to perform one of the best among many different modeling methods^18^. MaxEnt expresses the suitability of a grid cell as a function of the features(climatic layers) at that grid cell in a landscape, together with a set of occurrence records where the species has been observed. The MaxEnt suitability distribution is estimated by Equation 1:


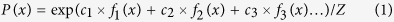


Here *c*_1_, *c*_2_, *c*_3_, … are constants, *f*_1_, *f*_2_, *f*_3_, … are the features, and *Z* is a scaling constant that ensures that *P* sums to 1 over all grid cells. It requires only species presence data (no absence) and environmental variable (continuous or categorical) layers for a target study area^19^,^20^. We used a freely available MaxEnt software, version 3.3^47^, which generates an estimate of probability of presence of the species that varies from 0 to 1, with 0 being the lowest probability and 1 being the highest. In this version of MaxEnt software, multivariate environmental similarity surface (MESS) analysis and limiting factors mapping (LFM) techniques have been integrated to explain the MaxEnt model outputs^21^. The value of MESS is calculated by Equation 2:


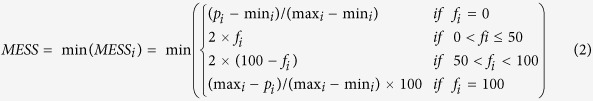


Here *min*_*i*_ is the minimum value of variable *i* over the reference point set and the similarly for *max*_*i*_. *p*_*i*_ is the value of variable *i* at a grid cell. *f*_*i*_ is the percent of reference points whose value of variable *i* is smaller than *p*_*i*_. MESS results allow the mapping of locations where limiting factors are important (LFM). The assumption is that information on which variable is driving the MESS value at any grid cell can be extracted and mapped by finding the variable with the smallest MESS value. MESS analysis explores whether there is possible novel habitat (extrapolation habitat) to inform the credibility of model output. LFM analysis brings insight into which climatic factors mostly limit physiological and ecological processes in each grid cell in target areas.

All occurrence records and 13 climatic predictors were uploaded in MaxEnt to model potential habitat distribution for oriental arborvitae. We use linear, quadratic, product, threshold, and hinge methods to generate feature types. The convergence threshold (10^−5^), maximum number of iterations (500), and 10,000 global background points were used to run the MaxEnt model. The logistic output was used to estimate the probability of presence (ranging from 0–1). A jackknife test (systematically leaving out each variable) and the regularized gain change [log of the number of grid cells minus the log loss (average of the negative log probabilities of the sample locations)] were then used to evaluate which climatic factors were the most important in determining the potential distribution of the species. The goodness-of-fit test of MaxEnt model is evaluated by the area under the threshold-independent receiver operating characteristic curve (AUC) based on 10-fold cross-validation method.

A suitable habitat map for oriental arborvitae was produced by utilizing the AUC weight averages of the 10 logistic output maps produced by 10-fold cross-validation, in which the relative suitability ranged from 0 to 1. Here, the probability threshold at which maximum test sensitivity plus specificity [max(*tp*/(*tp* + *fn*) + *tn*/(*tn* + *fp*)), *tp* is true positive value, *fn* is false negative vlaue, *fp* is false positive value and *tn* is true negative value] was selected as an optimal threshold. If the suitability value of a grid cell is greater than the optimal threshold, it will be considered as a potential distribution cell. We have calculated the optimal threshold of 10 logistic output maps in the simulation process of this study (range from 0.179–0.256 with mean value of 0.205 and standard deviation of 0.023). In order to preserve the maximum amount of forecast information and be convenient analysis in the next process, we divided the habitat suitability in the map into four levels: unsuitable habitat (0.0–0.2), marginal habitat (0.2–0.4), median habitat (0.4–0.6), and core habitat (0.6–1.0).

## Additional Information

**How to cite this article**: Li, G. *et al.* Mapping the climatic suitable habitat of oriental arborvitae (*Platycladus orientalis*) for introduction and cultivation at a global scale. *Sci. Rep.* **6**, 30009; doi: 10.1038/srep30009 (2016).

## Supplementary Material

Supplementary Information

## Figures and Tables

**Table 1:** Potential distribution countries of oriental arborvitae (probability threshold >0.2) around the world.

Region	Country	Climate Condition
Asia	China, Korea, Japan, India, Nepal, Iran, Georgia, Afghanistan, Tajikistan, Bhutan, Myanmar	Table S2
Europe	United Kingdom, France, Germany, Spain, Portugal, Italy, Czech Republic, Poland, Russia, Greece, Turkey, Hungary, Slovakia, Croatia, Yugoslavia, Romania, Ukraine, Belarus, Sweden, Denmark, Bulgaria, Albania	Table S3
North and Central America	United States, Mexico	Table S4
South America	Argentina, Chile, Brazil, Peru, Bolivia, Uruguay, Madagascar	Table S5
Africa	South Africa, Morocco, Angola, Ethiopia, Kenya, Zimbabwe	Table S6
Oceania	Australia, New Zealand	Table S7
