# Supplementary material for: Mapping the climatic suitable habitat of oriental arborvitae (Platycladus orientalis) for introduction and cultivation at a global scale
Source: Sci Rep. 2016 Jul 21;6:30009. doi: 10.1038/srep30009 (PMC4956768; doi:10.1038/srep30009)

Scientific Reports - Electronic Supplementary Material

Title: Mapping the climatic suitable habitat of oriental arborvitae (*Platycladus orientalis*) for introduction and cultivation at a global scale

Guoqing Li[1,2,*], Sheng Du[1,2], Zhongming Wen[1,2]

[1] State Key Laboratory of Soil Erosion and Dryland Farming on the Loess Plateau, Northwest A&F University, Yangling 712100, China

[2] Institute of Soil and Water Conservation, Chinese Academy of Sciences and Ministry of Water Resources, Yangling 712100, China

Corresponding Author: Guoqing Li

State Key Laboratory of Soil Erosion and Dryland Farming on the Loess Plateau, Northwest A&F University, Yangling 712100, China

Tel.: +86-29-87012411

Fax: +86-29-87012210

E-Mail: liguoqing@nwsuaf.edu.cn

Table S1 Climatic ranges of oriental arborvitae in the suitable habitat map as predicted by the MaxEnt model around the world

Climatic factor	Unit	Relative importance %	Climatic suitable habitat map			
			Core area 0.6-1.0	Medium area 0.4-0.6	Marginal area 0.2-0.4	Unsuitable area 0.0-0.2
CI	°C	27.1	-41.7-0.0	-57.2-0.0	-56.5-0.0	-328.2-0.0
WI	°C	21.9	54.0-235.8	30.6-259.7	16.1-294.3	0-369.9
PWM	mm	16.3	62-736	44-797	1-1200	1-1728
PSD	%	7.9	8-143	5-139	6-149	4-261
AMT	°C	6.6	3.2-20.1	-0.2-22.1	-2.1-25.0	-26.9-31.3
AP	mm	5.0	411-3272	147-3579	1-5214	1-8088
ART	°C	4.8	19.6-47.4	10.7-49.0	9.0-50.4	5.4-72.4
MTWM	°C	2.8	16.2-34.6	13.5-35.5	11.2-39.9	-5.9-48.8
PDM	mm	2.8	1-78	0-236	0-363	0-492
MTCM	°C	2.5	-20.7-6.5	-21.8-12.6	-22.6-19.5	-54.7-25.6
HI	mm /°C	1.6	2.3-20.1	1.2-31.2	0.0-50.5	0.0-100.0
ABT	°C	0.5	4.7-20.1	2.8-22.1	1.7-25.0	0.0-29.4
PER	°C /mm	0.2	0.2-2.1	0.1-3.9	0.1-109.0	0.0-1508.6

Coldness index (CI), warmth index (WI), precipitation of wettest month (PWM), precipitation of seasonality (PSD), annual mean temperature (AMT), annual precipitation (AP), annual range of temperature (ART), precipitation of driest month (PDM), mean temperature of the warmest month (MTWM), mean temperature of the coldest month (MTCM), humidity index (HI), annual biotemperature (ABT), potential evapotranspiration rate (PER).

Table S2 Climatic ranges of oriental arborvitae in the suitable habitat map as predicted by the MaxEnt model in Asia countries

Climatic factor	Unit	Relative importance %	Climatic suitable habitat map			
			Core area 0.6-1.0	Medium area 0.4-0.6	Marginal area 0.2-0.4	Unsuitable area 0.0-0.2
CI	°C	27.1	-41.7-0.0	-57.2-0.0	-56.5-0.0	-282.6-0.0
WI	°C	21.9	54.0-235.8	30.6-259.7	24.8-294.3	0.0-369.7
PWM	mm	16.3	96-736	44-797	8-1200	1-1728
PSD	%	7.9	36-143	7-139	6-149	7-168
AMT	°C	6.6	3.2-20.1	-0.2-22.1	-2.1-25.0	-22.3-31.3
AP	mm	5.0	411-3272	147-3579	30-4900	4-6458
ART	°C	4.8	21.3-47.4	11.9-49.0	9.3-50.4	7.2-72.4
MTWM	°C	2.8	16.7-34.5	13.5-34.8	12.1-39.9	2.0-46.4
PDM	mm	2.8	1-78	0-236	0-363	0-419
MTCM	°C	2.5	-20.7-6.5	-21.8-11.4	-22.6-19.5	-54.7-23.8
HI	mm /°C	1.6	2.6-19.2	1.2-21.1	0.1-27.2	0.0-100.0
ABT	°C	0.5	4.7-20.1	2.8-22.1	2.2-25.0	0.0-29.0
PER	°C /mm	0.2	0.2-1.8	0.2-3.9	0.1-25.3	0.0-337.3

Coldness index (CI), warmth index (WI), precipitation of wettest month (PWM), precipitation of seasonality (PSD), annual mean temperature (AMT), annual precipitation (AP), annual range of temperature (ART), precipitation of driest month (PDM), mean temperature of the warmest month (MTWM), mean temperature of the coldest month (MTCM), humidity index (HI), annual biotemperature (ABT), potential evapotranspiration rate (PER).

Table S3 Climatic ranges of oriental arborvitae in the suitable habitat map as predicted by the MaxEnt model in Europe countries

Climatic factor	Unit	Relative importance %	Climatic suitable habitat map			
			Core area 0.6-1.0	Medium area 0.4-0.6	Marginal area 0.2-0.4	Unsuitable area 0.0-0.2
CI	°C	27.1	0.0-0.0	-13.7-0.0	-21.9-0.0	-189.6-0.0
WI	°C	21.9	107.7-178.8	78.3-195.5	60.0-211.0	0.0-216.5
PWM	mm	16.3	62-227	52-210	50-210	21-342
PSD	%	7.9	8-50	8-85	8-83	7-87
AMT	°C	6.6	9.4-15.4	6.3-16.7	4.2-18.0	-15.3-18.5
AP	mm	5.0	662-1650	558-1396	428-1636	192-2754
ART	°C	4.8	21.3-29.3	17.0-33.0	13.1-34.7	11.8-51.2
MTWM	°C	2.8	21.3-31.2	18.4-31.6	13.9-34.6	1.9-36.0
PDM	mm	2.8	29-49	2-70	1-93	0-121
MTCM	°C	2.5	-0.9-1.9	-9.6-6.7	-10.8-8.4	-31.6-9.4
HI	mm /°C	1.6	6.1-9.3	3.5-14.1	2.2-20.0	0.0-96.5
ABT	°C	0.5	9.4-15.4	6.9-16.7	5.3-18.0	0.0-18.5
PER	°C /mm	0.2	0.5-0.8	0.3-1.4	0.2-2.2	0.0-3.8

Coldness index (CI), warmth index (WI), precipitation of wettest month (PWM), precipitation of seasonality (PSD), annual mean temperature (AMT), annual precipitation (AP), annual range of temperature (ART), precipitation of driest month (PDM), mean temperature of the warmest month (MTWM), mean temperature of the coldest month (MTCM), humidity index (HI), annual biotemperature (ABT), potential evapotranspiration rate (PER).

Table S4 Climatic ranges of oriental arborvitae in the suitable habitat map as predicted by the MaxEnt model in North and Central America countries

Climatic factor	Unit	Relative importance %	Climatic suitable habitat map			
			Core area 0.6-1.0	Medium area 0.4-0.6	Marginal area 0.2-0.4	Unsuitable area 0.0-0.2
CI	°C	27.1	-3.0-0.0	-26.5-0.0	-46.6-0.0	-328.2-0.0
WI	°C	21.9	76.2-214.4	65.5-253.2	49.3-278.7	0.0-340.8
PWM	mm	16.3	91-297	62-709	37-738	9-630
PSD	%	7.9	44-111	8-117	6-116	6-130
AMT	°C	6.6	6.8-18.3	5.2-21.6	1.5-23.7	-26.9-28.9
AP	mm	5.0	423-1843	266-3465	191-3750	54-4140
ART	°C	4.8	19.6-39.6	16.1-46.8	14.8-47.4	12.2-56.7
MTWM	°C	2.8	20.8-34.6	21.6-35.5	17.4-37.4	-5.9-43.5
PDM	mm	2.8	1-57	1-110	0-128	0-166
MTCM	°C	2.5	-8.6-5.0	-15.6-12.6	-20.9-14.9	-46.1-21.1
HI	mm /°C	1.6	2.3-20.1	1.4-31.2	1.1-38.4	0.0-100.0
ABT	°C	0.5	6.8-18.3	5.7-21.6	4.3-23.7	0.0-28.8
PER	°C /mm	0.2	0.2-2.1	0.1-3.5	0.1-4.5	0.0-24.1

Coldness index (CI), warmth index (WI), precipitation of wettest month (PWM), precipitation of seasonality (PSD), annual mean temperature (AMT), annual precipitation (AP), annual range of temperature (ART), precipitation of driest month (PDM), mean temperature of the warmest month (MTWM), mean temperature of the coldest month (MTCM), humidity index (HI), annual biotemperature (ABT), potential evapotranspiration rate (PER).

Table S5 Climatic ranges of oriental arborvitae in the suitable habitat map as predicted by the MaxEnt model in South America countries

Climatic factor	Unit	Relative importance %	Climatic suitable habitat map			
			Core area 0.6-1.0	Medium area 0.4-0.6	Marginal area 0.2-0.4	Unsuitable area 0.0-0.2
CI	°C	27.1	0.0-0.0	-5.2-0.0	-10.9-0.0	-47.1-0.0
WI	°C	21.9	61.4-184.9	33.4-236.0	16.1-274.9	0.7-342.0
PWM	mm	16.3	102-298	77-451	1-783	1-966
PSD	%	7.9	51-106	5-113	6-133	8-232
AMT	°C	6.6	5.6-15.9	3.2-20.1	1.2-23.4	-3.1-29.0
AP	mm	5.0	418-1944	327-2933	1-5214	2-8088
ART	°C	4.8	20.9-30.3	10.7-32.3	9.0-33.7	6.2-33.8
MTWM	°C	2.8	16.2-29.7	13.8-33.4	11.2-34.6	7.6-36.8
PDM	mm	2.8	2-62	1-128	0-283	0-492
MTCM	°C	2.5	-11.6-4.3	-10.9-10.8	-13.6-16.6	-14.4-23.2
HI	mm /°C	1.6	3.5-17.5	2.1-21.7	0.0-50.5	0.0-82.3
ABT	°C	0.5	5.6-15.9	3.2-20.1	1.7-23.4	0.1-28.9
PER	°C /mm	0.2	0.2-1.4	0.2-2.3	0.1-1013.5	0.0-595.1

Coldness index (CI), warmth index (WI), precipitation of wettest month (PWM), precipitation of seasonality (PSD), annual mean temperature (AMT), annual precipitation (AP), annual range of temperature (ART), precipitation of driest month (PDM), mean temperature of the warmest month (MTWM), mean temperature of the coldest month (MTCM), humidity index (HI), annual biotemperature (ABT), potential evapotranspiration rate (PER).

Table S6 Climatic ranges of oriental arborvitae in the suitable habitat map as predicted by the MaxEnt model in Africa countries

Climatic factor	Unit	Relative importance %	Climatic suitable habitat map			
			Core area 0.6-1.0	Medium area 0.4-0.6	Marginal area 0.2-0.4	Unsuitable area 0.0-0.2
CI	°C	27.1	0.0-0.0	0.0-0.0	-1.8-0.0	0.0-0.0
WI	°C	21.9	140.2-197.8	80.1-235.5	71.7-270.8	135.0-362.9
PWM	mm	16.3	141-265	70-401	19-442	1-1041
PSD	%	7.9	67-88	45-114	11-141	12-261
AMT	°C	6.6	12.1-16.9	7.1-20.1	6.4-23.0	11.7-30.7
AP	mm	5.0	853-1107	436-1935	57-2639	1-4159
ART	°C	4.8	21.1-26.5	12.1-37.1	10.9-38.7	9.5-42.7
MTWM	°C	2.8	22.8-27.8	17.2-33.7	16.4-35.7	21.8-48.8
PDM	mm	2.8	9-23	1-69	0-65	0-158
MTCM	°C	2.5	-2.3-6.1	-4.9-11.2	-8.3-13.2	-3.9-22.6
HI	mm /°C	1.6	4.9-7.0	2.0-14.5	0.2-13.9	0.0-13.9
ABT	°C	0.5	12.1-16.9	7.1-20.1	6.4-23.0	11.7-29.4
PER	°C /mm	0.2	0.7-1.0	0.3-2.4	0.3-16.8	0.3-1508.6

Coldness index (CI), warmth index (WI), precipitation of wettest month (PWM), precipitation of seasonality (PSD), annual mean temperature (AMT), annual precipitation (AP), annual range of temperature (ART), precipitation of driest month (PDM), mean temperature of the warmest month (MTWM), mean temperature of the coldest month (MTCM), humidity index (HI), annual biotemperature (ABT), potential evapotranspiration rate (PER).

Table S7 Climatic ranges of oriental arborvitae in the suitable habitat map as predicted by the MaxEnt model in Oceania countries

Climatic factor	Unit	Relative importance %	Climatic suitable habitat map			
			Core area 0.6-1.0	Medium area 0.4-0.6	Marginal area 0.2-0.4	Unsuitable area 0.0-0.2
CI	°C	27.1	0.0-0.0	0.0-0.0	-2.4-0.0	-6.9-0.0
WI	°C	21.9	157.0-189.1	94.0-235.7	66.0-268.7	48.0-338.9
PWM	mm	16.3	172-202	61-282	55-538	16-540
PSD	%	7.9	43-78	10-86	9-104	9-132
AMT	°C	6.6	13.5-16.2	8.3-20.1	5.8-22.8	3.9-28.7
AP	mm	5.0	845-1127	540-1985	411-2776	130-5207
ART	°C	4.8	23.5-26.3	17.8-29.6	14.4-31.0	9.5-34.3
MTWM	°C	2.8	24.8-31.4	19.0-33.2	17.6-34.5	15.9-41.7
PDM	mm	2.8	11-43	6-65	4-139	0-331
MTCM	°C	2.5	1.3-5.7	-2.9-9.1	-4.7-12.7	-6.0-22.0
HI	mm /°C	1.6	4.8-7.0	2.6-15.4	2.0-19.1	0.5-56.0
ABT	°C	0.5	13.5-16.2	8.3-20.1	5.9-22.8	4.3-28.0
PER	°C /mm	0.2	0.7-1.0	0.3-1.9	0.2-2.4	0.0-9.8

Coldness index (CI), warmth index (WI), precipitation of wettest month (PWM), precipitation of seasonality (PSD), annual mean temperature (AMT), annual precipitation (AP), annual range of temperature (ART), precipitation of driest month (PDM), mean temperature of the warmest month (MTWM), mean temperature of the coldest month (MTCM), humidity index (HI), annual biotemperature (ABT), potential evapotranspiration rate (PER).

Figure S1. Test and training AUC values of ten-fold cross-validation models (1-10 represent the model code, ascending in order by test AUC value; the mean test AUC is 0.918 and the mean training AUC value is 0.935).

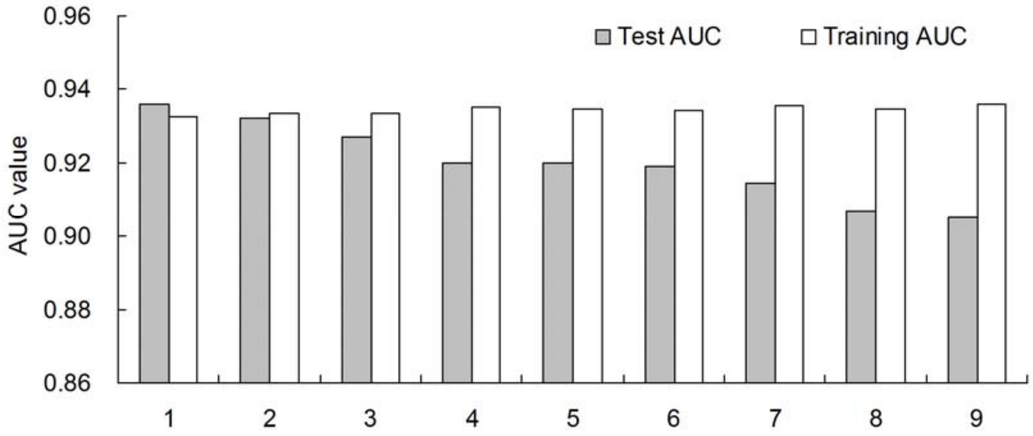

Figure S2. The relative importance of climatic factors (descending in order). Coldness index (CI), warmth index (WI), precipitation of wettest month (PWM), precipitation of seasonality (PSD), annual mean temperature (AMT), annual precipitation (AP), annual range of temperature (ART), precipitation of driest month (PDM), mean temperature of the warmest month (MTWM), mean temperature of the coldest month (MTCM), humidity index (HI), annual biotemperature (ABT), potential evapotranspiration rate (PER).

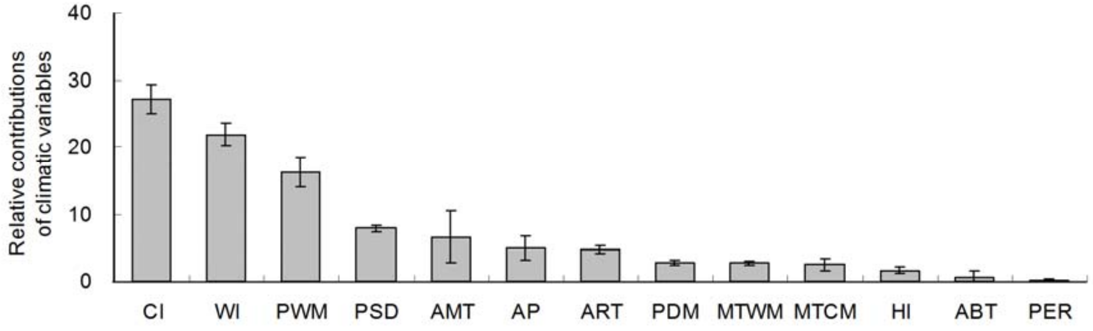

Supplement: Supplementary Information [file srep30009-s1.pdf]
